# Rice Bran as a Sustainable Feed Material Substitute to Improve the Meat Quality in Late-Stage Wenchang Chicken

**DOI:** 10.3390/biology15191686

**Published:** 2026-09-22

**Authors:** Jie Liu, Limin Wei, Qiqi Guo, Jingli Yuan, Yan Zhang, Xiuping Wang, Quanwei Liu

**Affiliations:** 1 Hainan Key Laboratory of Tropical Animal Breeding and Epidemic Research, Institute of Animal Husbandry & Veterinary Research, Hainan Academy of Agricultural Sciences, Haikou 571100, China; jieliu2303@163.com (J.L.);; 2 Sanya Institute, Hainan Academy of Agricultural Sciences, Sanya 572025, China; 3 Key Laboratory of Wenchang Chicken Breeding and Feeding, Hainan (Tan Niu) Wenchang Chicken Co., Ltd., Haikou 571100, China

**Keywords:** rice bran, feed substitute, yellow-feathered breed, Wenchang chicken, meat quality

## Abstract

The growing competition between humans and livestock for corn-soybean based feed calls for sustainable alternatives like rice bran. This study assessed substituting feed with rice bran (0%, 5%, 10%, 15%, 20%) in diets for slow-growing Wenchang chickens, a prized Chinese yellow-feathered breed. Up to 15% substitution maintained final body weight and average daily gain, but 20% substitution reduced body weight. Feed intake rose significantly at 10–20% substitution. Substituting 15–20% rice bran for feed significantly reduced abdominal fat without affecting slaughter performance or serum biochemical indicators. Meat quality had trade-offs: thigh muscle redness improved at 15%, and breast muscle fibers changed, but water-holding capacity decreased with increased cooking loss. Breast muscle intramuscular fat and inosinic acid increased, while fatty acid profiles changed, reducing both beneficial (e.g., oleic acid) and undesirable trans fatty acids at ≥10%, and saturated fatty acids in thigh muscle at 15%. Moreover, in the 20% group, alanine and glycine levels in breast muscles were notably lower than in the control and 5% groups. Additionally, the 15–20% group showed a significant reduction in total amino acids, the 10–20% group showed a significant reduction in flavor amino acids, and various free amino acids in thigh muscles were also reduced compared to the control group. Rice bran affected meat quality and nutrition, with mixed effects. Notably, incorporating a 10% rice bran substitute in the diet can improve meat quality traits in slow-growing yellow-feathered broiler chickens without impacting production performance.

## Simple Summary

This study explored using rice bran, a locally abundant by-product of rice processing, as a sustainable partial substitution for traditional corn and soybean feed in the final 40-day fattening stage of Wenchang chickens, a famous slow-growing yellow-feathered breed from Hainan. We tested five different substitution ratios from 0% to 20%. The results showed that up to 15% substitution maintained normal final body weight and average daily gain, while a 20% addition significantly reduced growth performance. The 15–20% substitution groups notably reduced abdominal fat deposition without impairing slaughter or serum biochemical health indicators. Meat quality showed mixed responses: 15% rice bran improved thigh muscle redness and breast muscle inosinic acid content, yet water-holding capacity, muscle fatty acid content, and amino acid content decreased at higher ratios. The 10% substitution level proved the optimal threshold, which improved key meat quality traits without any negative impact on production performance, offering a practical, low-cost feeding strategy for local yellow-feathered broiler production.

## 1. Introduction

With the rapid global population growth and significant increase in meat product demand, the competition for food between humans and livestock has become increasingly severe [1]. Traditional livestock production requires large amounts of corn and soybean meal as feed, which not only exacerbates food resource shortages but also puts considerable pressure on the environment [2]. Therefore, finding alternatives to corn and soybean meal in animal feed has become an urgent issue that needs immediate attention.

Corn and soybean meal are vital feed components due to their energy and nutritional benefits for livestock and poultry. Studies indicate that their digestibility significantly affects animal growth [3,4]. However, rising global demand for animal protein has driven up their prices, posing an economic challenge for breeders [5]. Rice bran is a nutrient-rich, unconventional energy feed with up to 16% crude protein (post-defatting) and a balanced amino acid profile [6], making it a superior plant protein source compared to traditional corn feed.

Research indicated that rice bran offers nutritional benefits and diverse applications. Rice bran protein (RBP), extracted from defatted rice bran using low heat, shows superior emulsifying and foaming properties compared to soy protein, highlighting its potential in the food industry [7]. Research showed that rice bran, due to its higher fat and balanced amino acids, is more energy-efficient for pig feed than corn bran or corn germ meal [8]. Its nutritional quality rivals that of animal and other plant proteins, confirming rice bran as a high-quality protein source [9]. The application of rice bran in animal feed has also shown significant advantages. Rice bran is abundant in natural antioxidants like gamma glutamylin, tocopherols, and plant sterols, offering health benefits and potential use in functional foods and supplements [10]. Gamma oryzanol shows strong antioxidant, anti-inflammatory properties with promising applications in health areas such as wound healing [11] and neuroprotection [12]. Rice bran tocopherol (vitamin E) supports antioxidant activity and acts as a drug delivery carrier [13], while its plant sterols reduce LDL-c and total cholesterol to improve blood lipids and prevent cardiovascular diseases [14].

Current research on rice bran in poultry primarily targets white-feathered broilers. Adding 10% rice bran to their feed promotes growth but reduces carcass quality [15], while over 20% hinders growth [16]. Another study indicates that a 5% rice bran substitution has no impact, but a 10% substitution significantly stunts growth [17], and substituting part of the diet with rice bran (15% and 30%) consistently reduced the final weight, weight gain, and FCR of Arbor Acres broiler chickens [18]. In addition, fermenting rice bran boosts its nutritional value, increasing crude protein and fat, which enhances animal growth and intestinal health [19,20]. The focus has been on the growth performance of white-feathered broiler chickens, with limited research on their slaughter performance and meat flavor.

The yellow-feathered broiler, a distinctive local chicken breed in China, is popular for its tasty meat, characterized by high intramuscular fat and excellent texture [21]. Wenchang chicken, a local breed from Hainan Province, China, is renowned for its quality meat and adaptability to tropical climates. It takes 110–120 days to market, with slow growth improving flavor but lowering feed efficiency. Thus, finding alternative feed materials to replace corn is essential [22]. Up to now, the use of rice bran as an unconventional feed for yellow-feathered broilers has not been thoroughly studied. Given the significance of yellow-feathered broilers in China’s animal husbandry and their distinct production and meat quality traits, this study focused on Wenchang chicken, a typical slow-growing yellow-feathered broiler, to investigate its potential as an alternative feed material by substituting feed with rice bran.

## 2. Materials and Methods

### 2.1. Test Material

Full-fat rice bran, obtained from a rice mill in Yongfa Town, Chengmai County, Hainan Province, China, was analyzed and found to contain 68.44 g/kg of crude protein, 4.46% crude fat, 36.75% crude fiber, 0.89 g/kg calcium, and 3.94 g/kg available phosphorus. Detection methods followed the Chinese national standards GB/T 18868-2024 (China, 2024), GB/T 6437-2018 (China, 2018), and GB/T 8573-2017 (China, 2017).

### 2.2. Animal Experiment

The study used Wenchang hens bought from Hainan Tanniu Wenchang Chicken Co., Ltd., Haikou, Hainan Province, China. A total of 500 70-day-old Wenchang chicken hens, with similar genetic backgrounds and body weight (1152.50 g ± 1.53), were randomly divided into five treatment groups. Each group consisted of five replicates, with 20 hens per replicate. The control group (0%) received only the basal diet, while the four experimental groups were fed diets in which 5%, 10%, 15%, or 20% of the feed was replaced with rice bran. The basal diet was formulated according to the “Nutrition Requirement of Yellow Feather Broiler” (NY/T3645-2020) using corn and soybean meal. All diets were formulated to maintain consistent levels of metabolizable energy, key amino acids, and crude protein. The composition and nutritional levels of the experimental diets are presented in Table 1. The feeding trial was conducted at the Yongfa Experimental Base of the Hainan Academy of Agricultural Sciences (Chengmai County, Hainan Province, China). Birds were housed in cages (65 cm long, 45 cm wide, and 35 cm high) with free access to feed and water and were exposed to a combination of natural and artificial light. Routine immunization procedures were followed. The experiment lasted 40 days. At the end of the trial, birds were electrically stunned and euthanized by exsanguination in accordance with the 2020 AVMA Guidelines, followed by weighing. All experimental procedures were reviewed and approved by the Experimental Animal Ethics Committee of the Animal Husbandry and Veterinary Research Institute, Hainan Academy of Agricultural Sciences (Approval No. HNSYY20240802, dated 2 August 2024).

### 2.3. Growth Performance

Feed intake, initial body weight (IBW), and final body weight (FBW) were recorded for each replicate throughout the experiment. Average daily feed intake (ADFI), average daily gain (ADG), and feed conversion ratio (FCR) were subsequently calculated.

### 2.4. Slaughtering Performance

At the conclusion of the experiment, two birds per replicate were randomly selected, weighed, and euthanized via exsanguination. Carcass weight, semi-eviscerated weight, fully eviscerated weight, breast muscle weight, and thigh muscle weight were measured following the guidelines of “Poultry Production Performance Terminology and Measurement Statistical Method” (NY/T823/2020) [23]. Corresponding percentages, including dressing percentage, semi-eviscerated percentage, eviscerated percentage, breast muscle percentage, thigh muscle percentage, and abdominal fat percentage, were calculated.

### 2.5. Organ Index

Following slaughter, the heart, liver, spleen, and stomach were excised. Excess moisture and blood were removed using filter paper, and adhering tissues and fat were trimmed before weighing. Organ indices were calculated using the following formula: organ indices (%) = organ weight/live body weight × 100.

### 2.6. Serum Biochemical Indicators

Blood samples were collected from the wing vein into procoagulant tubes and centrifuged at 2500× *g* for 15 min. The supernatant was collected and stored at −80 °C for subsequent analysis. Serum biochemical parameters, including total protein (TP), albumin (ALB), globulin (GLB), alkaline phosphatase (ALP), aspartate aminotransferase (AST), triglycerides (TG), low-density lipoprotein cholesterol (LDL-c), high-density lipoprotein cholesterol (HDL-c), glucose, and uric acid, were measured using kits from the Nanjing Jiancheng Institute of Biological Engineering (Nanjing, Jiangsu Province, China), following the manufacturer’s protocols.

### 2.7. Meat Quality and Nutrient Content

To evaluate meat quality, skinless whole breast and thigh meat were collected from slaughtered birds. Measurements of pH, drip loss, cooking loss, cooked meat rate, and shear force of breast muscles, as well as the determination of moisture, crude protein, intramuscular fat, inosinic acid, fatty acids, and amino acids in both breast and thigh muscles, were conducted following previously described methods [24].

### 2.8. Breast and Thigh Muscle Morphology

Right breast and thigh muscle samples were fixed in 4% paraformaldehyde. Samples were sectioned longitudinally using a rotary microtome (Leica RM2255 microtome, Heerbrugg, Switzerland). Muscle tissue morphology was examined using hematoxylin and eosin (H&E) staining preceded by standard protocol. The morphology of the muscle tissue was observed using a fluorescence microscope (Olympus XM10, Olympus, Japan). The number and total area of muscle fibers per section were measured using ImagePro Plus 6.0 software, and fiber diameter, cross-sectional area, and density were calculated.

### 2.9. Fatty Acid Contents in Breast and Thigh Muscle

Left breast and thigh meat samples were stored at −20 °C and subsequently analyzed for fatty acid composition using ultra-high performance liquid chromatography (LC20, Shimadzu, Kyoto, Japan) in accordance with GB 5009.168-2016. Fatty acid types and contents were determined based on fatty acid methyl ester content and conversion coefficients.

### 2.10. Amino Acid Contents in Breast and Thigh Muscle

Following the method described by Qi et al. [25], approximately 30 mg of freeze-dried meat samples were hydrolyzed in 6 M hydrochloric acid at 110 °C for 24 h. The hydrolysate was diluted with distilled water, and 1 mL of the diluted solution was filtered through a 0.2 µm membrane filter. Amino acid analysis was performed using an HPLC-based amino acid analyzer (Ultimate3000-API 3200 Q TRAP; ThermoFisher Scientific, Foster City, CA, USA) according to the manufacturer’s instructions. Amino acid content in muscle tissue was expressed as μg per mL of fresh meat. TAA content was calculated as the arithmetic sum of all 18 individual amino acids quantified via the pre-column derivatization HPLC method. EAA was calculated as the sum of 8 amino acids that are classified as nutritionally indispensable for broilers: threonine, valine, methionine, isoleucine, leucine, phenylalanine, lysine and tryptophan. The EAA/TAA ratio was further derived by dividing total EAA content by the corresponding TAA value for each sample. Sweet amino acids were summed from the subset of flavor-related amino acids with confirmed sweet taste characteristics in poultry meat sensory studies, including glycine, alanine, serine, threonine, lysine and proline. Umami amino acids were calculated as the sum of aspartic acid and glutamic acid.

### 2.11. Statistical Analysis

Data are presented as mean ± standard error of the mean (SEM) for figures or mean ± standard deviation (SD) for tables. Differences were statistically analyzed with One-way analysis of variance (ANOVA) using SAS software (version 8.02, SAS Institute, Cary, NC, USA). Differences were considered statistically significant at *p* < 0.05 and highly significant at *p* < 0.01.

## 3. Results

### 3.1. The Growth Performance of Wenchang Chicken

As shown in Figure 1, compared to the control group, using 5–15% rice bran in place of feed did not impact FBW (*p* > 0.05), but 20% did reduce it (*p* < 0.01). The ADFI was significantly higher in the 10%, 15%, and 20% groups compared to the control group (*p* < 0.01), with no significant difference between the control and 5% groups (*p* > 0.05). The 20% group had a significantly higher FCR than the control, 5%, and 10% groups (*p* < 0.01), while the control, 5%, 10%, and 15% groups showed no significant differences (*p* > 0.05). The five groups showed similar average daily weight gain (*p* > 0.05).

### 3.2. The Slaughter Performance of Wenchang Chicken

Figure 2 indicates no significant differences among the five groups in dressing percentage, half-eviscerated yield percentage, eviscerated yield percentage, thigh muscle percentage, and breast muscle percentage (*p* > 0.05). The 15% group showed a significant reduction in abdominal fat percentage compared to the control, 5%, and 10% groups, while the 20% group had a significant reduction compared to the control and 10% groups (*p* < 0.01). There were no significant differences in abdominal fat among the control, 5%, and 10% groups (*p* > 0.05).

Figure 2 shows that the stomach index of the 15% group was significantly higher than the control group (*p* < 0.05), but not significantly different from the 5% and 10% groups (*p* > 0.05). No significant differences were observed in the heart, liver, and spleen indices across all groups (*p* > 0.05).

### 3.3. The Serum Biochemical Indicators of Wenchang Chicken

Figure 3 shows that substituting feed with rice bran does not significantly affect the serum biochemical indexes of Wenchang chickens (*p* > 0.05).

### 3.4. Meat Quality and Muscle Morphology

Figure 4 shows that the 5% and 15% groups had significantly higher cooking loss in breast and thigh muscles than the control group (*p* < 0.01), while the 10% group showed no significant difference (*p* > 0.05). The 20% group had significantly higher cooking loss in thigh muscles compared to the control (*p* < 0.05). Moreover, the 15% group showed a significant improvement in thigh muscle redness compared to the other four groups (*p* < 0.01). The five groups showed no notable differences in other physical and chemical indicators (*p* > 0.05).

As shown in Figure 5, the 20% group had a significantly smaller breast muscle diameter, decreasing by 22.02%, 15.53% and 15.01% relative to the control, 5% and 15% groups, while the 10% group was 12.67% smaller than the control. No significant differences were found among the control, 5%, and 15% groups (*p* < 0.01). The 5% group had a significantly larger average cross-sectional area of breast muscles than the 10%, 15%, and 20% groups, but not compared to the control group (*p* < 0.05). Rice bran substitution did not affect the diameter, cross-sectional area, or density of thigh muscles (Figure 5E–H, *p* > 0.05).

### 3.5. Nutrient Composition

Figure 6 indicates that the 5–20% group notably reduced breast muscle moisture, with the 10% group showing the greatest reduction. The five groups showed no notable differences in crude protein (*p* > 0.05). The intramuscular fat content in the 10% and 20% groups rose slightly compared to the control group, but the increase was not statistically significant (*p* = 0.07). Additionally, the 15% and 20% groups had significantly higher inosinic acid compared to the control, whereas the 5% and 10% groups did not show a significant difference. In thigh muscle (Figure 6E–H), the 5–15% group had significantly higher moisture, and the 5–20% group significantly reduced crude protein content compared to the control group, with the 20% group showing the largest decrease (*p* < 0.01). The five groups had similar levels of intramuscular fat (*p* > 0.05). Rice bran substitution did not alter creatine content compared to the control group; however, the 15% and 20% groups had significantly higher levels than the 10% group, and the 15% group exceeded the 5% group.

### 3.6. Muscle Fatty Acids in Wenchang Chicken

In breast muscles (Table 2), the 10–20% groups showed a reduction in unsaturated fatty acids compared to the control group. Specifically, the 10%, 15%, and 20% groups had lower levels of myristic acid (C14:0), oleic acid (C18:1N9C), trans oleic acid (C18:1N9T), trans linoleic acid (C18:2N6T), alpha linolenic acid (C18:3N3), dihigh-γ-linoleic acid (C20:3N6), and eicosapentaenoic acid (C20:5N3), while the 10% and 15% groups also had reduced decanoic acid (C10:0) and pentadecanoic acid (C15:0).

In thigh muscles (Table 3), the 15% group had significantly reduced levels of saturated and unsaturated fatty acids, decanoic acid (C10:0), myristic acid (C14:0), pentadecanoic acid (C15:0), and palmitic acid (C16:0) compared to the control. The 20% group had lower C14:0, and the 10% group had higher γ-linoleic acid (C18:3N6) and docosahexaenoic acid (C22:6N3) levels than the control.

### 3.7. Muscle Amino Acids in Wenchang Chicken

Table 4 indicates that rice bran substitution did not significantly impact the levels of total amino acids (TAA), essential amino acids (EAA), EAA/TAA ratio, sweet amino acids, and umami amino acids compared to the control group in breast muscles (*p* > 0.05). Moreover, in the breast muscles, alanine and glycine levels in the 20% group were significantly lower than in the control and 5% groups, with no significant difference from other groups (*p* > 0.05). In the thigh muscles (Table 5), the TAA content was significantly lower in the 15–20% group compared to the control (*p* < 0.01), while the sweet amino acid content was significantly lower in the 10–20% group compared to both the control and 5% groups (*p* < 0.01). The alanine, glycine, and valine levels in the 10%, 15%, and 20% groups were significantly lower than in the control group (*p* < 0.05). Lysine in the 20% group and leucine, isoleucine, and phenylalanine in the 15% and 20% groups were also significantly lower than in the control group (*p* < 0.05).

## 4. Discussion

Finding suitable corn alternatives has promising potential, particularly in China, to alleviate food competition between humans and livestock. Yellow-feathered broilers occupy a significant position in China’s poultry industry, with large production volumes but longer growth cycles, particularly for slow-growing yellow-feathered broilers. Wenchang chicken is the largest single breed of yellow-feathered broilers in China. Rice bran is a nutrient-rich feed, high in protein, dietary fiber, amino acids, vitamins B, minerals, and essential fatty acids for poultry [19]. Research indicated that substituting 5% rice bran for feed did not affect the growth performance of white-feathered broilers, but the impact is minimal due to the low substitution rate [17]. However, rice bran shows greater potential as a feed substitute in yellow-feathered broilers, particularly those with longer growth cycles. Our study found that substituting rice bran at levels below 15% did not affect the growth of Wenchang chickens but did increase ADFI (Figure 1), and it had no impact on serum biochemical indicators. The increase in ADFI is consistent with the rise in stomach index. We speculate that the insoluble dietary fiber in rice bran increased the volume of intestinal contents, thereby accelerating intestinal peristalsis and the feed passage rate [26]. In addition, the non-starch polysaccharides in rice bran also reduced nutrient digestion efficiency [27], leading to the observed increases in both feed intake and FCR. In addition, the increased fiber content in rice bran as a substitute for feed may also lead to this situation.

Slaughter performance serves as a crucial indicator for evaluating meat production efficiency. Our research showed that substituting rice bran in the diet reduced abdominal fat in Wenchang chickens (Figure 2D) without impacting other slaughter metrics. Accumulating abdominal fat in animals impacts growth and meat quality, with higher belly fat leading to more fat content and less lean meat, affecting taste and nutrition [28]. It also reduces meat’s oxidative stability, impacting shelf life and flavor [29]. The decrease in abdominal fat may result from rice bran’s higher crude fiber content compared to corn, which enhances digestion and boosts feed intake [30].

There was no significant difference in serum biochemical indicators, likely because rice bran substitution feed has a minimal impact on these indicators in slow-growing Wenchang chickens. This may be due to the nutritional similarity between rice bran and corn, which prevents significant metabolic or physiological disturbances with moderate substitution. Additionally, chickens can self-regulate and adapt to feed changes, maintaining stable serum biochemical indicators. Further research is needed to explore the specific mechanisms involved.

Meat color is a crucial sensory indicator for assessing meat quality, as consumers often judge freshness and quality based on appearance [31]. Myoglobin’s redox state significantly impacts meat color, affecting consumer acceptance and purchasing decisions [32]. Our research showed that 15% and 20% rice bran enhance thigh muscle redness without altering breast muscle color. We hypothesize that the enhanced redness of thigh muscles is likely due to bioactive compounds in rice bran [11], like gamma oryzanol and tocopherol, which may boost antioxidant activity by scavenging free radicals, preventing lipid oxidation, and protecting myoglobin, thereby maintaining a brighter red color.

Furthermore, the impact of rice bran on meat quality extends beyond color. Muscle fiber diameter, area, and density were deemed as key elements for water-holding capacity of muscle [33]. Higher muscle fiber density and diameter result in more tender meat [34]. The study revealed that the breast muscle fiber diameter was significantly smaller in the 20% and 10% groups compared to the control group. Muscle density increased in the 15% and 20% groups, though not significantly. These changes in muscle fiber characteristics may contribute to improved meat texture and juiciness. Moreover, we discovered that substituting feed with rice bran significantly decreased the moisture in breast muscles while increasing it in thigh muscles, impacting the chicken’s water retention ability. Based on the results, substituting 10% with rice bran may enhance breast muscle tenderness and water retention, while higher or lower levels may hinder these effects.

The crude protein, intramuscular fat, and inosinic acid content in chicken are crucial factors affecting meat quality [35]. Studies show that adjusting feed composition and additives can significantly improve both nutritional value and product quality. The nutritional analysis showed that in the breast muscle, both beneficial and harmful fatty acids decreased, but intermuscular fat and creatine levels rose, enhancing meat quality with rice bran substitution. Conversely, in the thigh muscles, creatine increased, but moisture and crude protein significantly decreased, with no change in intermuscular fat, resulting in a decline in meat quality.

Fatty acids play a crucial role in determining the flavor profile of meat, including chicken, where both saturated and unsaturated fats contribute significantly to the sensory attributes [36]. The composition of fatty acids in meat can influence its taste, aroma, and overall palatability, which are essential factors for consumer acceptance and preference. The study investigated the effects of dietary incorporation of linseed oil and soybean isoflavone on the fatty acid profiles of chicken breast muscle. The results indicated that the inclusion of linseed oil significantly increased the content of n-3 polyunsaturated fatty acids while reducing saturated fatty acids in the meat. This alteration in fatty acid composition not only improved the nutritional value of the chicken meat but also potentially enhanced its flavor profile by increasing the levels of beneficial unsaturated fats [37]. Similarly, the inclusion of safflower oil in chicken diets was found to increase the levels of linoleic acid and omega-3 fatty acids in the breast muscle, which are known to contribute positively to meat flavor and health benefits [38]. Although the fat content of the rice bran substituted diet will gradually increase due to the inherent fat contained in rice bran, feeding with the rice bran-substituted diet, especially at the 15% substitution levels, can significantly reduce the contents of both saturated and unsaturated fatty acids in breast and thigh muscles. These results imply that while moderate rice bran substitution can influence fatty acid profiles, the specific impact varies depending on the level of substitution. Moreover, based on the analysis of muscle nutritional components and fatty acid testing results, rice bran substitution improves breast muscle meat quality but reduces leg muscle meat quality.

The amino acid composition directly influences both flavor profile and nutritional value of chicken. Research demonstrates that adjusting feed formulations can significantly modify amino acid levels in poultry. For instance, substituting fermented soybean meal for conventional soybean meal partially enhances glutamic acid and total free amino acid content, thereby improving meat’s antioxidant properties and moisture retention capacity [39]. Our study found that substituting 5–10% of the diet with rice bran did not significantly change the composition of amino acids in the breast and thigh muscles of Wenchang chickens. And further research is needed to fully understand the mechanisms behind these alterations.

Our research focused on the 70–110-day-old Wenchang chicken, with a focus on rice bran as a feed substitute. We recognize this is a limitation of the current study that may have potential confounding effects on the observed results. We have added this point to Section 4 of the manuscript to clarify the possible impact of unadjusted crude fiber content on the research conclusions, and we will control this variable more strictly in follow-up related studies. Additionally, this article lacks analysis of gut microbiota and metabolites, which are crucial for understanding their impact on growth performance.

## 5. Conclusions

In conclusion, our study has demonstrated that substituting 10–15% rice bran for feed in Wenchang chicken diets increased ADFI without compromising growth. Meat quality improved with redder thigh muscles and changed breast muscle fibers, but water-holding capacity decreased. Replacing 5–10% rice bran has a relatively small effect on muscle amino acid composition, but it increases intramuscular fat and inosinic acid in breast muscles, decreases crude protein in thigh muscle, and 10–15% substitution reduces both beneficial and harmful fatty acids in breast muscle. These findings suggest that rice bran affects meat quality and nutrition, with mixed effects. Notably, incorporating a 10% rice bran substitute in the diet can improve meat quality traits in slow-growing yellow-feathered broiler chickens without impacting production performance.

## Figures and Tables

**Figure 1:**
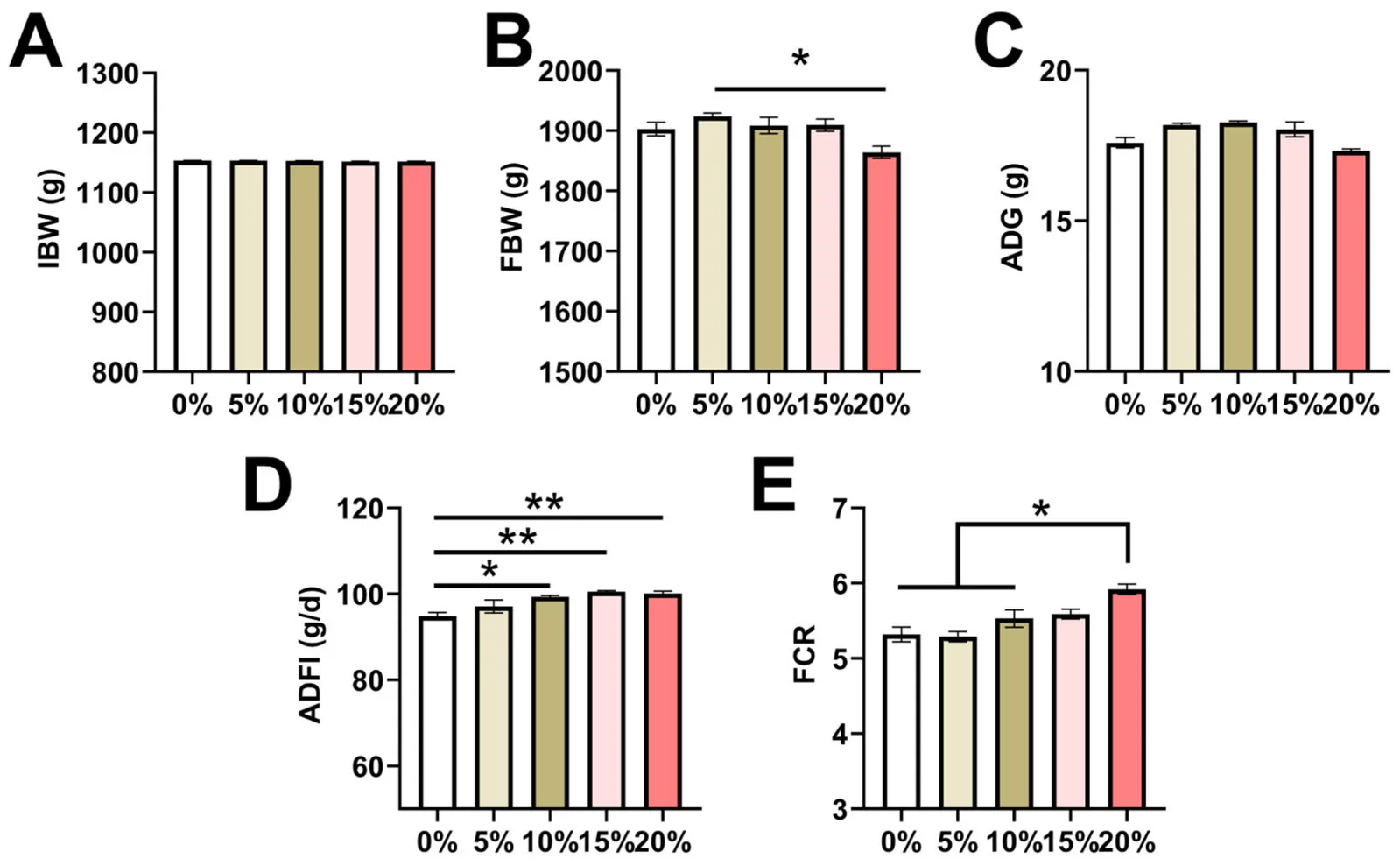
Effects of different substitution levels of rice bran on growth performance of Wenchang chickens. (**A**–**E**) The initial body weight (IBW, (**A**)), final body weight (FBW, (**B**)), average daily gain (ADG, (**C**)), average daily feed intake (ADFI, (**D**)), and feed conversion ratio (FCR, (**E**)) of Wenchang chickens. Data are presented as the mean ± SEM. * *p* < 0.05 is considered significant and ** *p* < 0.01 is considered highly significant, as determined by a one-way ANOVA analysis. The number of samples in each group is ten.

**Figure 2:**
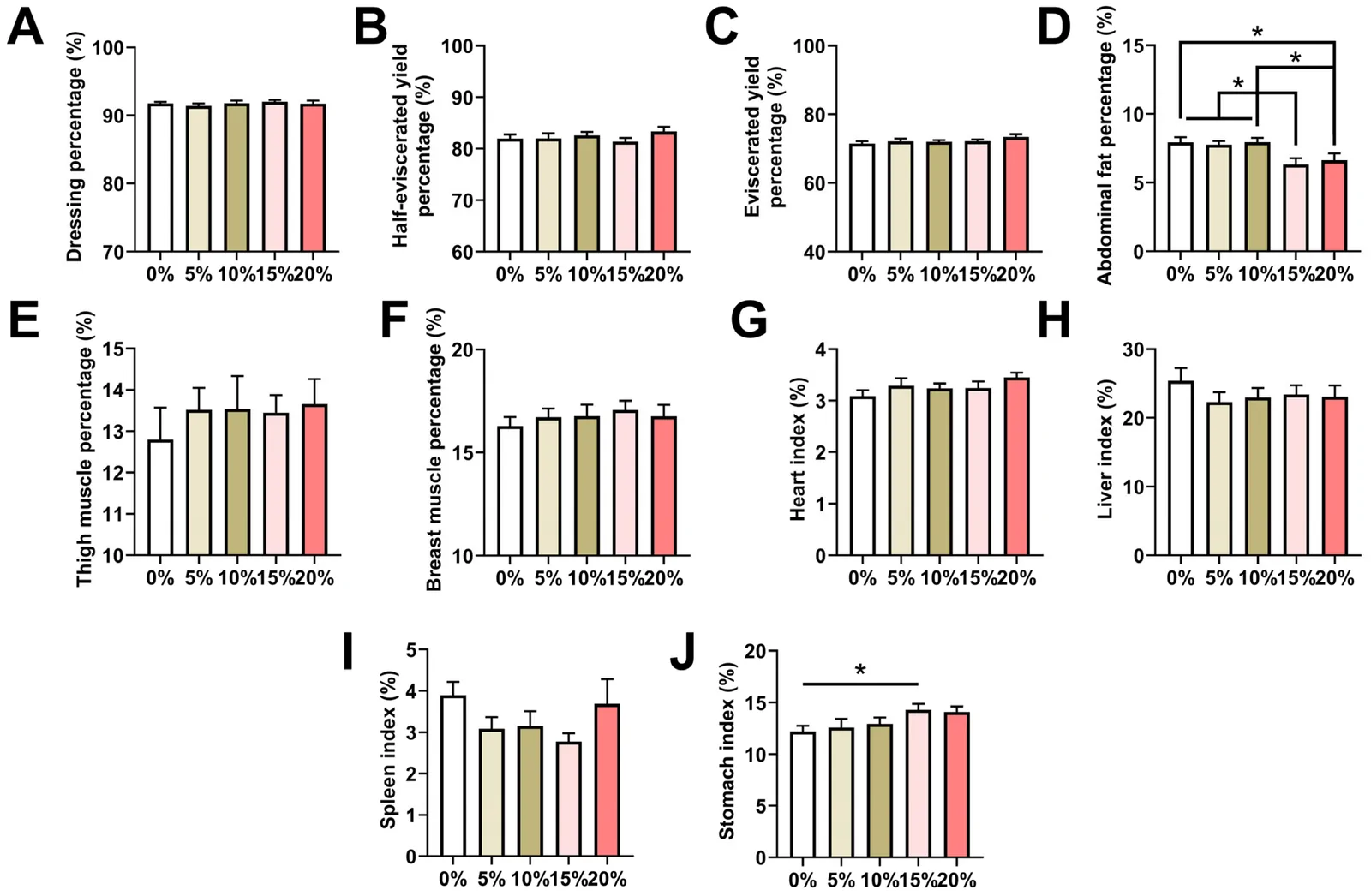
Effects of different substitution levels of rice bran on slaughter performance of Wenchang chickens. (**A**–**F**) The dressing percentage (**A**), half-eviscerated yield percentage (**B**), eviscerated yield percentage (**C**), abdominal fat percentage (**D**), thigh muscle percentage (**E**), and breast muscle percentage (**F**) of Wenchang chickens. (**G**–**J**) The organ index of Wenchang chickens. Different superscript letters indicate significant differences. Data are presented as the mean ± SEM. * *p* < 0.05 is considered significant, as determined by a one-way ANOVA analysis. The number of samples in each group is ten.

**Figure 3:**
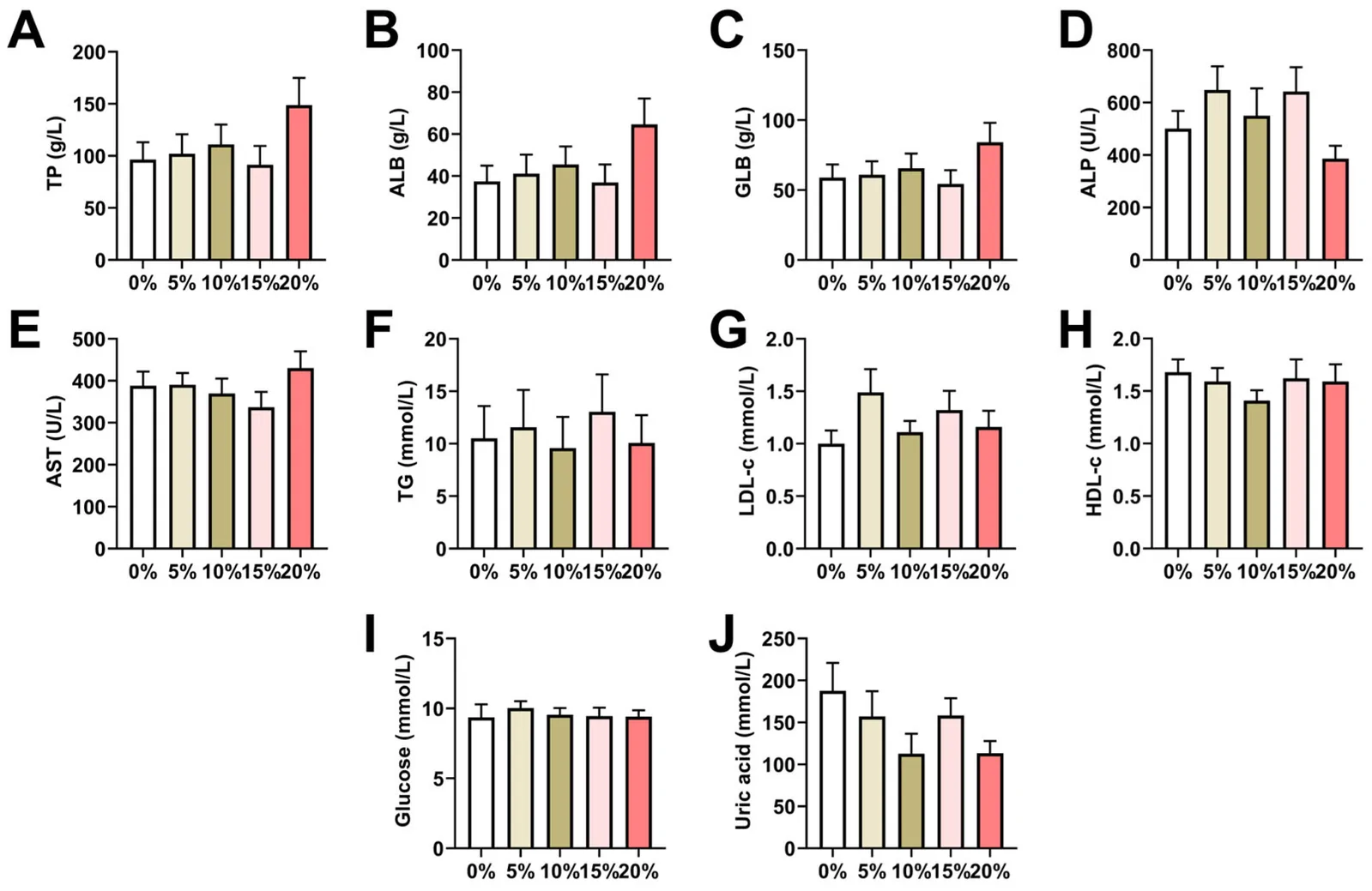
The serum biochemical indicators of Wenchang chicken. The total protein (TP, (**A**)), albumin (ALB, (**B**)), globulin (GLB, (**C**)), alkaline phosphatase (ALP, (**D**)), aspartate aminotransferase (AST, (**E**)), triglycerides (TG, (**F**)), low density lipoprotein (LDL-c, (**G**)), high-density lipoprotein (HDL-c, (**H**)), glucose (**I**), and uric acid (**J**) in serum. Data are presented as the mean ± SEM. The number of samples in each group is ten.

**Figure 4:**
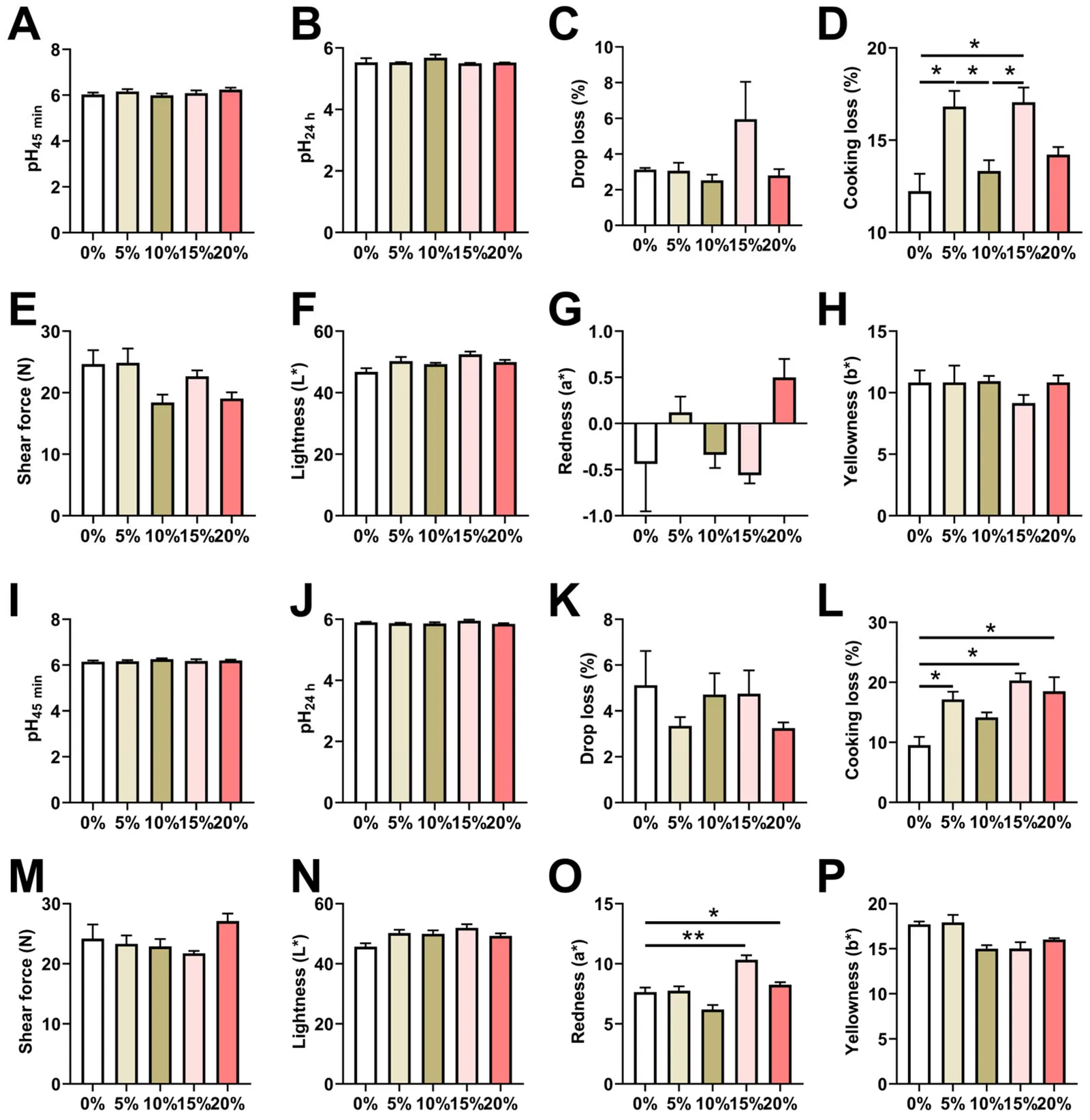
The meat quality of Wenchang chicken. (**A**–**H**) The pH after 45 min (pH _45min_, (**A**)), pH after 24 h (pH _24h_, (**B**)), drop loss (**C**), cooking loss (**D**), shear force (**E**), lightness (**F**), redness (**G**), and yellowness (**H**) in breast muscle. (**I**–**P**) The pH after 45 min (**I**), pH after 24 h (**J**), drop loss (**K**), cooking loss (**L**), shear force (**M**), lightness (**N**), redness (**O**), and yellowness (**P**) in thigh muscle. Data are presented as the mean ± SEM. * *p* < 0.05 is considered significant and ** *p* < 0.01 is considered highly significant, as determined by a one-way ANOVA analysis. The number of samples in each group is ten.

**Figure 5:**
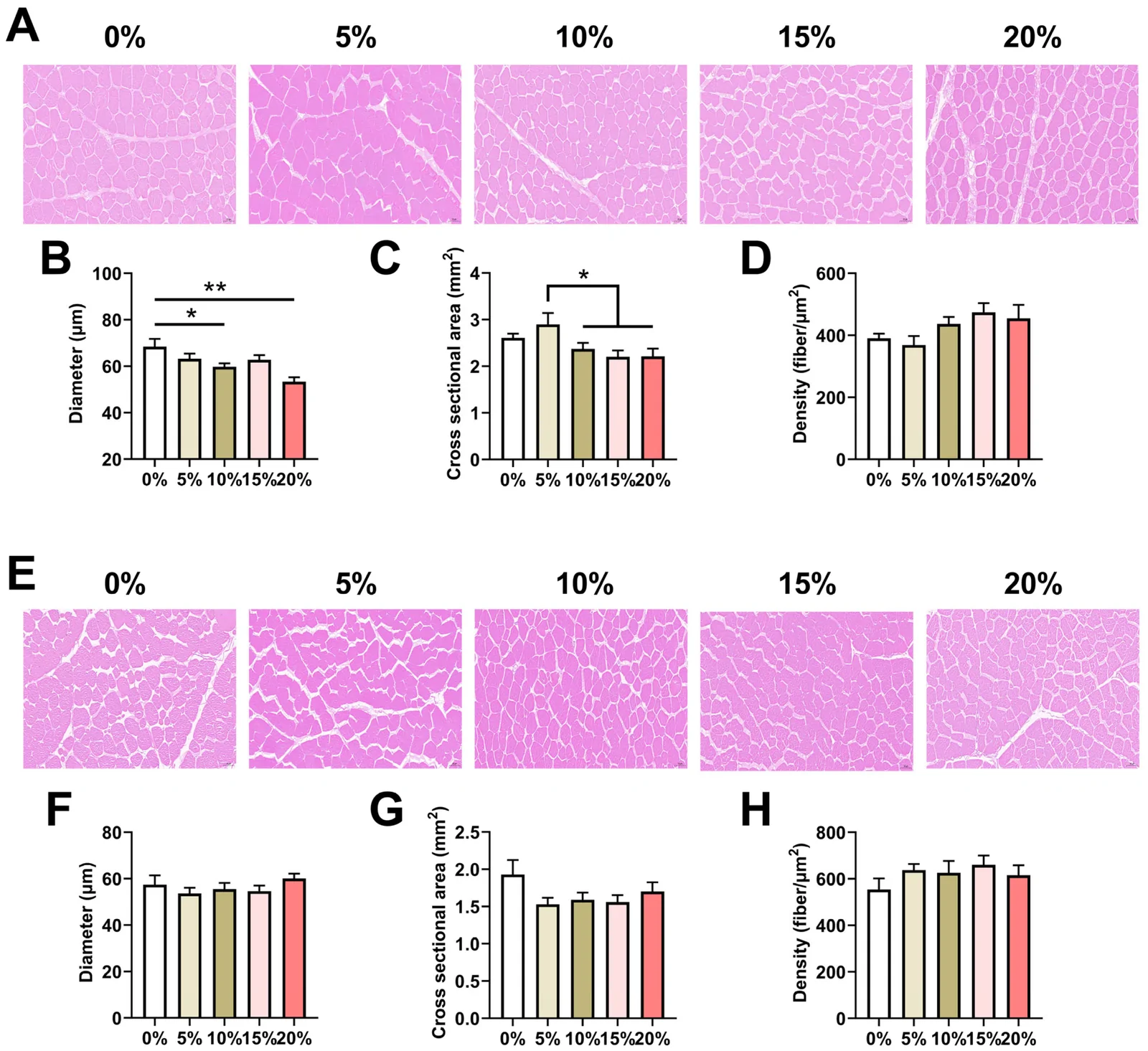
The muscle morphology. (**A**) Morphologic analysis of breast muscles in five groups. Scale bars: 50 µm. (**B**–**D**) The diameter (**B**), cross sectional area (**C**), and density (**D**) were calculated. (**E**) Morphologic analysis of thigh muscles in five groups. Scale bars: 50 µm. (**F**–**H**) The diameter (**F**), cross sectional area (**G**), and density (**H**) were calculated. Data are presented as the mean ± SD. * *p* < 0.05 is considered significant and ** *p* < 0.01 is considered highly significant, as determined by a one-way ANOVA analysis. The number of samples in each group is ten.

**Figure 6:**
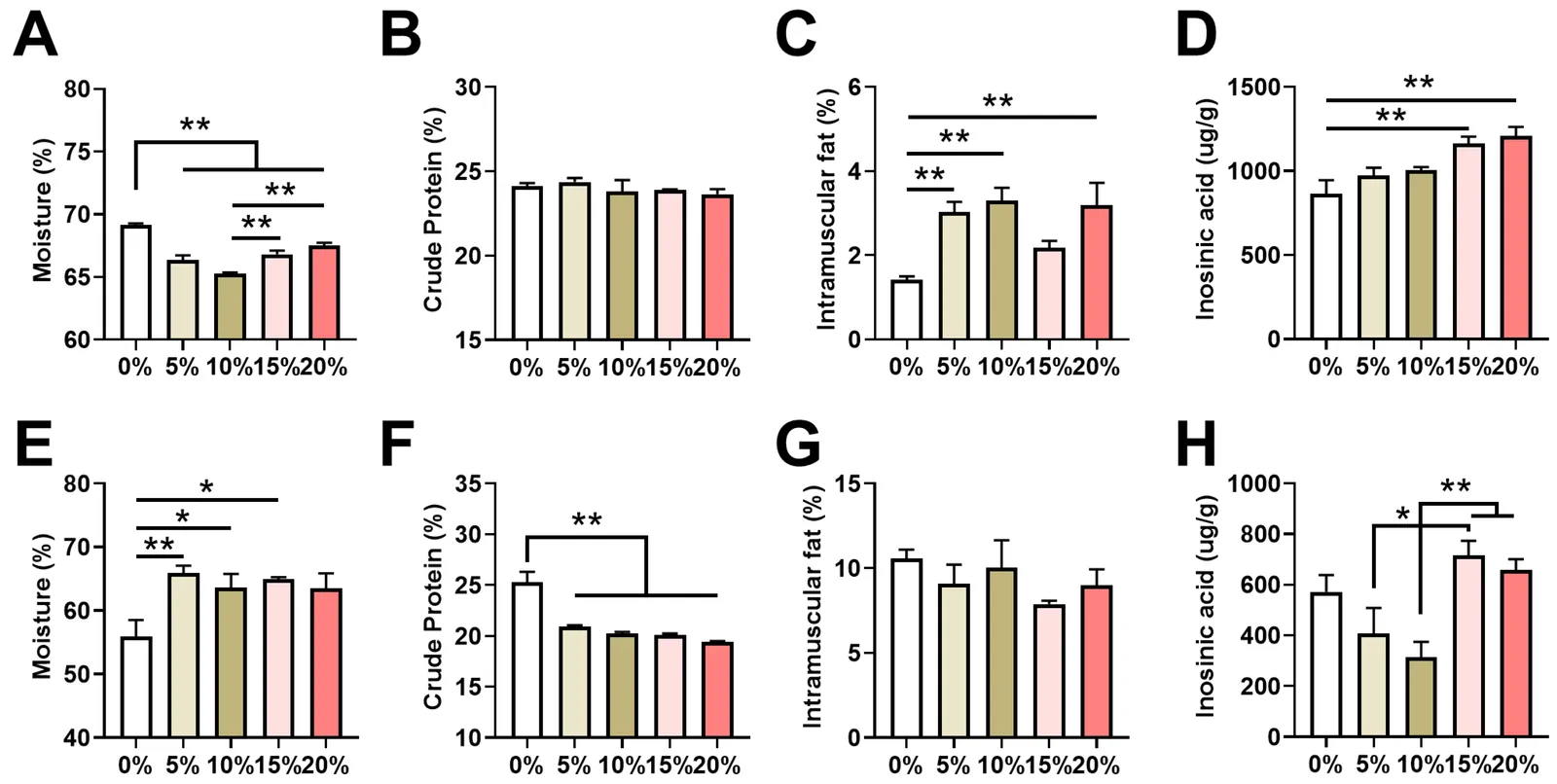
The nutrient composition in muscle. (**A**–**D**) The concentration of moisture (**A**), crude protein (**B**), intramuscular fat (**C**), and inosinic acid (**D**) in breast muscle. (**E**–**H**) The concentration of moisture (**E**), crude protein (**F**), intramuscular fat (**G**), and inosinic acid (**H**) in thigh muscle. Data are presented as the mean ± SEM. * *p* < 0.05 is considered significant and ** *p* < 0.01 is considered highly significant, as determined by a one-way ANOVA analysis. The number of samples in each group is ten.

**Table 1:** Composition and nutrient level of feeds (dry matter basis, %).

Ingredients Composition (%)	0%	5%	10%	15%	20%
Corn	69.57	64.47	59.49	54.48	49.46
Rice bran	0.00	5.00	10.00	15.00	20.00
Soybean meal	16.95	16.55	16.09	15.66	15.22
Fish meal	2.00	2.00	2.00	2.00	2.00
Bran	5.00	5.00	5.00	5.00	5.00
Vegetable oil	3.00	3.50	3.95	4.41	4.88
Stone powder	0.96	0.98	0.98	0.98	0.98
CaHPO_4_	0.93	0.93	0.93	0.93	0.93
NaCl	0.30	0.30	0.30	0.30	0.30
L-lysine HCl	0.10	0.08	0.07	0.06	0.05
DL-methionine	0.09	0.09	0.09	0.08	0.08
Choline chloride	0.10	0.10	0.10	0.10	0.10
Premix ^1^	1.00	1.00	1.00	1.00	1.00
Nutrient level ^2^ (dry mater basis, %)					
ME, MJ/kg	12.74	12.74	12.74	12.74	12.74
Crude protein	15.83	15.83	15.83	15.83	15.83
Crude fiber	5.30	8.40	10.08	13.27	13.00
Crude Fat	6.13	7.26	8.33	9.42	10.52
Calcium	0.79	0.79	0.79	0.79	0.79
Available phosphorus	0.36	0.36	0.36	0.36	0.36
Lysine	0.85	0.85	0.85	0.85	0.85
Methionine	0.36	0.36	0.36	0.36	0.36
Methionine + Cystine	0.64	0.64	0.64	0.62	0.62

^1^ The premix provided the following per kg of feed: Cu 10 mg, Fe 80 mg, Mn 60 mg, Zn 70 mg, I 2 mg, Se 0.40 mg, vitamin A 10000 IU, vitamin D 32000 IU, vitamin E 10 mg, vitamin B1 2 mg, vitamin B2 3 mg, vitamin B6 3.5 mg, nicotinic acid 15 mg, folic acid 0.50 mg, pantothenic acid 10 mg, biotin 0.15 mg, and cyanocobalamin 10 μg. ^2^ Nutrient levels were calculated values, and the nutritional value data of ingredients were referred to in the Table of Feed Composition and Nutritional Value in China (2024).

**Table 2:** Effects of different substitution levels of rice bran in feed on the fatty acid in Wenchang chicken breast muscle (μg/mL).

Traits	Groups					*p*-Value
	0%	5%	10%	15%	20%	
Saturated fatty acids	1589.78 ± 328.73	1577.18 ± 429.80	1169.64 ± 118.48	1148.66 ± 132.58	1391.64 ± 302.28	0.059
Unsaturated fatty acid	2075.12 ± 327.56 ^a^	1878.81 ± 356.63 ^ab^	1657.17 ± 170.92 ^b^	1621.60 ± 141.86 ^b^	1699.04 ± 148.18 ^b^	0.009
Polyunsaturated fatty acid	1960.71 ± 297.38	1807.75 ± 355.50	1563.07 ± 156.99	1536.51 ± 137.00	1687.39 ± 150.92	0.055
C10:0	3.91 ± 0.39 ^a^	3.96 ± 0.37 ^a^	3.47 ± 0.30 ^b^	3.47 ± 0.07 ^b^	3.71 ± 0.11 ^ab^	0.027
C12:0	7.84 ± 1.44	8.32 ± 2.38	6.22 ± 1.03	6.15 ± 0.20	7.04 ± 1.16	0.096
C13:0	4.31 ± 0.29	4.51 ± 0.84	4.34 ± 0.19	4.17 ± 0.23	4.48 ± 0.03	0.101
C14:0	47.06 ± 11.04 ^a^	44.08 ± 14.43 ^ab^	30.08 ± 14.25 ^bc^	20.85 ± 1.39 ^c^	28.11 ± 13.65 ^bc^	0.011
C15:0	9.37 ± 1.37 ^a^	8.95 ± 1.06 ^ab^	7.60 ± 0.49 ^b^	7.66 ± 0.45 ^b^	8.42 ± 1.12 ^ab^	0.034
C16:0	1084.70 ± 240.30	1073.00 ± 357.40	770.90 ± 78.32	751.90 ± 84.39	911.20 ± 213.14	0.068
C17:0	8.84 ± 1.84	8.91 ± 2.37	7.24 ± 0.44	7.23 ± 0.51	9.00 ± 2.06	0.225
C18:0	411.50 ± 72.04	414.60 ± 62.84	326.40 ± 29.59	332.70 ± 51.87	404.50 ± 73.21	0.058
C20:0	8.48 ± 0.51	8.23 ± 0.26	8.18 ± 0.37	7.97 ± 0.28	8.41 ± 0.59	0.368
C24:0	6.28 ± 0.36	5.87 ± 0.26	6.51 ± 0.73	6.56 ± 0.33	6.78 ± 0.63	0.223
C18:1N9C	176.40 ± 26.68 ^a^	151.90 ± 33.72 ^ab^	138.00 ± 20.10 ^b^	125.20 ± 14.99 ^b^	121.00 ± 13.71 ^b^	0.007
C18:1N9T	111.60 ± 20.74 ^a^	91.60 ± 22.08 ^ab^	82.70 ± 12.53 ^b^	81.50 ± 11.74 ^b^	88.40 ± 5.95 ^b^	0.045
C18:2N6	519.90 ± 67.69	479.20 ± 95.40	407.00 ± 50.28	404.50 ± 83.82	432.30 ± 33.87	0.071
C18:2N6T	84.60 ± 10.79 ^a^	72.30 ± 13.40 ^ab^	69.40 ± 8.97 ^ab^	64.10 ± 5.87 ^b^	62.30 ± 5.63 ^b^	0.010
C18:3N3	423.00 ± 69.94 ^a^	351.90 ± 88.55 ^ab^	319.20 ± 52.31 ^b^	289.50 ± 39.90 ^b^	280.10 ± 37.39 ^b^	0.009
C18:3N6	10.30 ± 0.76	9.68 ± 0.83	9.51 ± 0.40	9.09 ± 0.47	9.39 ± 0.53	0.092
C20:1	11.52 ± 1.13	10.43 ± 1.14	9.18 ± 0.30	9.29 ± 0.90	9.61 ± 1.67	0.069
C20:2	18.17 ± 1.49	17.71 ± 2.22	15.19 ± 1.35	16.12 ± 2.11	25.40 ± 14.16	0.169
C20:3N6	75.70 ± 8.80 ^a^	69.70 ± 6.70 ^ab^	57.10 ± 2.97 ^c^	62.96 ± 10.94 ^bc^	65.10 ± 3.19 ^bc^	0.008
C20:4N6	377.40 ± 88.15	391.10 ± 95.32	316.80 ± 21.86	338.70 ± 34.72	437.70 ± 60.64	0.076
C20:5N3	60.60 ± 7.30 ^a^	60.00 ± 2.69 ^ab^	50.07 ± 2.59 ^c^	52.74 ± 2.88 ^c^	55.00 ± 1.27 ^bc^	0.001
C22:6N3	105.10 ± 19.11	116.20 ± 27.79	98.10 ± 8.04	92.10 ± 6.04	110.70 ± 15.51	0.222

Data are presented as the mean ± SD. Different superscript letters indicate significant differences (*p* < 0.05). The number of samples in each group is five.

**Table 3:** Effects of different substitution levels of rice bran in feed on the fatty acid in Wenchang chicken thigh muscle (μg/mL).

Traits	Groups					*p*-Value
	0%	5%	10%	15%	20%	
Saturated fatty acid	1502.12 ± 111.34 ^a^	1345.22 ± 249.31 ^ab^	1671.18 ± 476.63 ^a^	1068.66 ± 263.11 ^b^	1361.32 ± 142.47 ^ab^	0.036
Unsaturated fatty acid	2120.08 ± 201.32 ^ab^	1981.16 ± 519.68 ^ab^	2574.45 ± 696.88 ^a^	1640.97 ± 377.10 ^b^	1922.19 ± 277.82 ^ab^	0.049
Polyunsaturated fatty acid	1962.71 ± 178.94	1824.20 ± 442.50	2409.84 ± 705.10	1540.74 ± 355.86	1908.91 ± 279.93	0.063
C10:0	3.88 ± 0.22 ^a^	3.82 ± 0.33 ^ab^	4.23 ± 0.51 ^a^	3.39 ± 0.26 ^b^	3.75 ± 0.30 ^ab^	0.017
C12:0	7.69 ± 0.76	7.52 ± 1.04	8.05 ± 2.92	5.86 ± 0.93	7.28 ± 1.07	0.254
C13:0	4.29 ± 0.27	4.09 ± 0.21	4.42 ± 0.24	4.18 ± 0.24	4.35 ± 0.20	0.206
C14:0	50.62 ± 6.22 ^a^	44.31 ± 15.48 ^ab^	55.60 ± 7.86 ^a^	21.54 ± 7.22 ^c^	31.79 ± 11.79 ^bc^	<0.001
C15:0	8.79 ± 0.57 ^a^	8.38 ± 0.76 ^a^	9.20 ± 0.99 ^a^	7.21 ± 0.91 ^b^	8.18 ± 0.45 ^ab^	0.007
C16:0	875.5 ± 88.51 ^a^	839.00 ± 154.07 ^a^	983.9 ± 240.05 ^a^	622.8 ± 146.70 ^b^	801.50 ± 75.52 ^ab^	0.02
C17:0	9.23 ± 0.74	8.51 ± 0.91	10.08 ± 2.50	7.74 ± 1.37	8.83 ± 1.05	0.175
C18:0	517.80 ± 75.02	414.70 ± 82.94	576.60 ± 239.87	379.30 ± 110.65	478.20 ± 71.87	0.172
C20:0	9.97 ± 0.47	9.13 ± 1.19	11.22 ± 2.48	9.21 ± 1.32	9.88 ± 0.93	0.191
C24:0	7.08 ± 0.74	7.20 ± 1.39	7.88 ± 1.23	7.44 ± 1.06	7.56 ± 0.52	0.762
C18:1N9C	163.60 ± 23.93	162.70 ± 49.98	200.50 ± 60.73	118.70 ± 28.81	148.80 ± 29.87	0.070
C18:1N9T	93.80 ± 8.47	93.80 ± 26.22	117.90 ± 33.85	78.80 ± 14.50	97.40 ± 19.71	0.139
C18:2N6T	82.70 ± 10.83 ^ab^	81.10 ± 21.10 ^ab^	98.40 ± 24.42 ^a^	62.24 ± 11.68 ^b^	75.60 ± 13.52 ^b^	0.049
C18:2N6	588.60 ± 74.16	532.70 ± 111.51	744.30 ± 298.72	473.10 ± 165.60	581.20 ± 127.01	0.193
C18:3N3	382.10 ± 60.11	380.70 ± 131.05	477.80 ± 157.02	267.10 ± 73.90	347.50 ± 77.25	0.073
C18:3N6	10.70 ± 0.41 ^bc^	10.96 ± 1.67 ^b^	12.46 ± 0.81 ^a^	9.36 ± 0.92 ^c^	10.15 ± 1.26 ^bc^	0.004
C20:1	11.67 ± 0.49	10.56 ± 1.82	13.13 ± 3.22	9.93 ± 1.11	11.14 ± 1.65	0.122
C20:2	19.35 ± 11.75	13.83 ± 0.83	16.64 ± 2.49	13.57 ± 1.30	15.01 ± 0.72	0.448
C20:4N6	438.10 ± 73.76	369.70 ± 109.43	520.50 ± 150.67	343.60 ± 86.01	433.10 ± 40.45	0.083
C20:5N3	52.75 ± 6.15	45.06 ± 13.72	55.30 ± 4.78	49.46 ± 3.19	52.76 ± 5.56	0.289
C20:3N6	52.21 ± 3.68	50.65 ± 4.41	52.74 ± 3.78	49.21 ± 5.85	53.69 ± 2.95	0.497
C22:6N3	78.80 ± 9.77 ^b^	83.00 ± 17.23 ^b^	113.30 ± 23.85 ^a^	75.60 ± 10.64 ^b^	93.70 ± 19.67 ^ab^	0.015

Data are presented as the mean ± SD. Different superscript letters indicate significant differences (*p* < 0.05). The number of samples in each group is five.

**Table 4:** Effects of different substitution levels of rice bran in feed on the amino acid in Wenchang chicken breast muscle (mg/kg).

Traits ^1^	Groups					*p*-Value
	0%	5%	10%	15%	20%	
TAA	1825.53 ± 174.64	1927.44 ± 354.42	1660.91 ± 185.91	1658.01 ± 276.31	1637.57 ± 220.18	0.302
EAA	1093.44 ± 114.68	1205.49 ± 339.66	1012.83 ± 151.34	1025.15 ± 208.57	1012.93 ± 110.03	0.523
EAA/TAA(%)	59.87 ± 2.22	61.77 ± 6.17	60.77 ± 2.43	61.48 ± 4.46	62.97 ± 2.93	0.906
Sweet amino acids	411.94 ± 42.33	424.84 ± 28.35	363.21 ± 38.40	368.54 ± 43.48	366.71 ± 82.74	0.202
Umami amino acids	219.37 ± 40.62	195.72 ± 45.73	194.99 ± 21.69	161.36 ± 47.42	166.01 ± 42.92	0.185
methionine	10.94 ± 4.99	11.68 ± 2.20	10.63 ± 4.84	5.79 ± 3.65	10.72 ± 10.27	0.533
arginine	36.66 ± 5.92	42.90 ± 9.76	37.28 ± 15.27	34.18 ± 12.77	45.92 ± 24.98	0.729
isoleucine	31.28 ± 6.39	28.86 ± 5.37	24.68 ± 4.75	18.64 ± 5.79	25.50 ± 18.02	0.312
Phenylalanine	33.14 ± 6.05	28.32 ± 2.82	25.38 ± 4.65	20.36 ± 7.07	28.90 ± 20.98	0.428
lysine	39.92 ± 19.06	44.82 ± 20.67	31.08 ± 15.16	29.46 ± 8.83	27.62 ± 14.10	0.401
leucine	16.50 ± 3.44	16.26 ± 4.01	12.86 ± 2.98	9.16 ± 3.15	13.14 ± 10.37	0.252
threonine	57.24 ± 11.35	53.80 ± 6.31	55.06 ± 11.13	48.52 ± 14.61	71.82 ± 47.22	0.603
valine	26.40 ± 7.37	31.40 ± 9.86	25.40 ± 6.50	22.20 ± 5.17	23.60 ± 15.18	0.608
histidine	877.80 ± 115.64	990.40 ± 318.88	827.80 ± 121.39	871.20 ± 184.00	811.20 ± 134.75	0.617
alanine	284.40 ± 40.76 ^ab^	292.00 ± 19.27 ^a^	240.80 ± 22.92 ^bc^	247.40 ± 28.47 ^bc^	222.20 ± 42.31 ^c^	0.011
glutamate	178.20 ± 38.02	159.00 ± 49.30	159.60 ± 23.13	125.14 ± 40.04	139.22 ± 41.18	0.283
cystine	89.32 ± 13.02	79.04 ± 10.66	76.52 ± 2.07	87.80 ± 6.41	82.28 ± 14.11	0.259
glycine	13.86 ± 2.28 ^a^	13.8 ± 1.26 ^a^	9.77 ± 1.83 ^b^	11.44 ± 2.09 ^ab^	10.24 ± 3.18 ^b^	0.019
proline	4.80 ± 4.37	13.72 ± 10.10	6.92 ± 1.96	12.89 ± 4.36	10.54 ± 3.79	0.092
serine	51.94 ± 13.32	51.78 ± 10.39	50.78 ± 6.38	48.12 ± 6.58	51.92 ± 12.84	0.972
tyrosine	32.00 ± 5.10	33.40 ± 4.34	31.40 ± 5.81	29.80 ± 5.97	35.60 ± 18.04	0.892
aspartate	41.34 ± 27.34	36.72 ± 7.12	35.58 ± 7.49	36.34 ± 16.52	26.78 ± 5.66	0.664

^1^ TAA, total amino acids; EAA, essential amino acids. Data are presented as the mean ± SD. Different superscript letters indicate significant differences (*p* < 0.05). The number of samples in each group is five.

**Table 5:** Effects of different substitution levels of rice bran in feed on the amino acid in Wenchang chicken thigh muscle (mg/kg).

Traits ^1^	Groups					*p*-Value
	0%	5%	10%	15%	20%	
TAA	3102.08 ± 659.11 ^a^	2854.00 ± 501.72 ^a^	2705.93 ± 538.23 ^ab^	2074.69 ± 335.40 ^bc^	1992.34 ± 215.53 ^c^	0.005
EAA	1100.08 ± 479.27	1195.66 ± 343.46	984.71 ± 319.58	830.89 ± 345.75	812.86 ± 283.32	0.398
EAA/TAA(%)	35.59 ± 11.98	42.96 ± 12.86	38.22 ± 15.86	40.65 ± 15.50	40.63 ± 12.11	0.931
Sweet amino acids	850.24 ± 259.33 ^a^	828.29 ± 179.88 ^a^	596.30 ± 106.57 ^b^	523.85 ± 124.16 ^b^	477.72 ± 96.19 ^b^	0.004
Umami amino acids	981.62 ± 616.00	676.55 ± 776.73	981.49 ± 707.21	566.37 ± 578.66	566.46 ± 364.00	0.683
Alanine	491.80 ± 173.14 ^a^	450.80 ± 96.19 ^ab^	335.80 ± 53.80 ^bc^	286.40 ± 76.59 ^c^	249.00 ± 56.69 ^c^	0.004
Arginine	135.20 ± 22.92	124.84 ± 28.96	114.14 ± 36.08	118.30 ± 26.10	107.72 ± 14.70	0.554
Aspartate	471.96 ± 250.32	248.38 ± 282.92	452.38 ± 368.02	216.74 ± 201.64	226.26 ± 169.24	0.353
Cystine	44.26 ± 15.46	45.28 ± 14.63	49.94 ± 19.52	42.00 ± 14.94	43.74 ± 13.42	0.946
Glutamate	509.80 ± 413.86	428.00 ± 496.31	529.00 ± 345.24	349.80 ± 379.98	340.20 ± 200.03	0.896
Lysine	127.54 ± 42.26 ^ab^	140.12 ± 40.32 ^a^	88.20 ± 24.79 ^bc^	92.54 ± 31.18 ^bc^	61.82 ± 18.61 ^c^	0.008
Glycine	103.22 ± 36.56 ^a^	124.72 ± 54.79 ^a^	41.44 ± 14.20 ^b^	44.48 ± 18.13 ^b^	30.62 ± 12.35 ^b^	<0.001
Histidine	575.60 ± 398.17	758.60 ± 382.03	612.00 ± 316.45	546.80 ± 326.48	550.80 ± 242.04	0.849
Leucine	62.62 ± 19.42 ^a^	46.78 ± 18.36 ^ab^	41.28 ± 10.92 ^bc^	23.42 ± 7.42 ^c^	24.90 ± 6.04 ^c^	<0.001
Isoleucine	99.62 ± 34.93 ^a^	77.30 ± 26.92 ^ab^	67.74 ± 15.34 ^bc^	41.36 ± 10.65 ^c^	42.52 ± 9.96 ^c^	0.002
Methionine	24.16 ± 14.60	12.31 ± 4.76	19.12 ± 13.64	8.95 ± 2.83	8.73 ± 5.01	0.074
Phenylalanine	74.74 ± 38.76 ^a^	54.10 ± 14.47 ^ab^	50.82 ± 13.37 ^ab^	32.86 ± 6.26 ^b^	34.92 ± 9.24 ^b^	0.024
Proline	16.31 ± 13.19	11.28 ± 12.57	6.23 ± 6.74	19.34 ± 13.45	11.88 ± 5.10	0.392
Serine	175.48 ± 73.95	186.80 ± 56.59	153.52 ± 59.74	129.02 ± 26.45	132.60 ± 30.30	0.351
Threonine	63.32 ± 31.07	54.64 ± 15.42	59.08 ± 15.77	44.36 ± 6.49	53.54 ± 15.50	0.585
Tyrosine	53.80 ± 22.56	38.20 ± 10.80	38.20 ± 9.18	37.60 ± 7.47	37.40 ± 7.44	0.225
Valine	72.40 ± 30.20 ^a^	51.80 ± 17.81 ^ab^	46.40 ± 11.99 ^b^	40.60 ± 12.20 ^b^	35.60 ± 5.41 ^b^	0.032

^1^ TAA, total amino acids; EAA, essential amino acids. Data are presented as the mean ± SD. Different superscript letters indicate significant differences (*p* < 0.05). The number of samples in each group is five.

## Data Availability

The original contributions generated for this study are included in the article. Further inquiries can be directed to the corresponding author.

## Abbreviations

The following abbreviations are used in this manuscript: LDL-c, low-density lipoprotein cholesterol; TC, total cholesterol; FCR, feed conversion ratio; IBW, initial body weight; FBW, final body weight; ADFI, average daily feed intake; ADG, average daily gain; TP, total protein; ALB, albumin; GLB, globulin; ALP, alkaline phosphatase; AST, aspartate aminotransferase; TG, triglycerides; HDL-c, high-density lipoprotein cholesterol; H&E, hematoxylin and eosin; HPLC, high-performance liquid chromatography; SEM, standard error of the mean; SD, standard deviation; ANOVA, analysis of variance; TAA, total amino acids; EAA, essential amino acids.
